# [Expression of Concern] Resveratrol improves neurological outcome and neuroinflammation following spinal cord injury through enhancing autophagy involving the AMPK/mTOR pathway

**DOI:** 10.3892/mmr.2025.13679

**Published:** 2025-09-10

**Authors:** Hong-Yu Meng, De-Cheng Shao, Han Li, Xiao-Dan Huang, Guang Yang, Bing Xu, Hai-Yun Niu

Mol Med Rep 18: 2237–2244, 2018; DOI: 10.3892/mmr.2018.9194

Following the publication of this paper, it was drawn to the Editor's attention by a concerned reader that, regarding the confocal microscopic images shown in Fig 3, the top (Sham) and bottom (SCI) data panels appeared to show a small section of overlapping data, such that data which were intended to show the results from differently performed experiments had apparently been derived from the same original source. The authors were contacted by the Editorial Office to offer an explanation for this apparent anomaly in the presentation of the data in this paper; however, up to this time, no response from them has been forthcoming. Owing to the fact that the Editorial Office has been made aware of potential issues surrounding the scientific integrity of this paper, we are issuing an Expression of Concern to notify readers of this potential problem while the Editorial Office continues to investigate this matter further.

